# Editorial: The Active Brain

**DOI:** 10.3389/fpsyg.2024.1385888

**Published:** 2024-03-12

**Authors:** Joseph M. Northey, Charles H. Hillman, Sabrina Skorski, Kristy Martin

**Affiliations:** ^1^Research Institute for Sport and Exercise, University of Canberra, Canberra, ACT, Australia; ^2^Discipline of Sport and Exercise Science, Faculty of Health, University of Canberra, Canberra, ACT, Australia; ^3^Center for Cognitive and Brain Health, Northeastern University, Boston, MA, United States; ^4^Department of Psychology, Northeastern University, Boston, MA, United States; ^5^Department of Physical Therapy, Movement, and Rehabilitation Sciences, Northeastern University, Boston, MA, United States; ^6^Institute of Sport and Preventive Medicine, Saarland University, Saarbrücken, Germany

**Keywords:** exercise, cognition, physical activity, sleep, brain health

## Introduction

The Active Brain represents a multidisciplinary field of research examining the interrelationships between physical activity and the brain across the contexts of health and performance. Understanding these interrelationships and considering the physical and mental aspects of an individual as a whole is essential for maximizing individuals' safety, wellbeing, and performance across various settings and throughout the lifespan. This Research Topic is a collection of articles that provide a greater understanding of the brain and its interaction with physical activity across health and human performance settings. Individually, each article advances the field by utilizing novel measures, interventions, and population groups in their protocols. As a collective, these articles highlight the diversity of this research field and the challenges in progressing evidence.

## Preview of the Research Topic

Quinlan et al. and Gajewski et al. highlight the complexity of investigating the role of physical activity in promoting cognitive and brain health across the lifespan. Aspects of cognition begin to decline from middle age, and while this stage of life is seen as a potentially sensitive period to increase cognitive reserve through engagement in physical activity, relatively little evidence is available compared to older age. Quinlan et al. used 7-day accelerometry in 156 middle-aged adults aged 37–43 to investigate relationships between physical activity and cognitive function. Despite investigating overall physical activity and a range of intensity cut points, the authors found no associations with cognition. Gajewski et al. extend the findings of Quinlan et al. in their study, which investigated whether age moderates the relationship between physical fitness, a proxy for engagement in physical activity, and cognition. In their sample of 490 participants aged 20–70, only the 46–70-year-old participants displayed multiple positive associations between fitness and cognitive function. The authors conclude that these findings suggest older participants benefit more from being physically fit, and that it is also likely that this age group displays sufficient heterogeneity in the trajectory of cognitive changes for any benefit of lifestyle to be identified. Gajewski et al. suggest in their title that this is an “advantage of being older”, however one could also posit that this is of course an advantage to being physically fit when older. These two studies are an important addition to the evidence around physical activity and brain health in middle-age and will undoubtedly aid in methodological advances to the field.

One of the challenges in this area is to increase the diversity of the populations engaged in producing evidence on the effects of physical activity on the brain. This Research Topic features four manuscripts contributing to such diversity by recruiting various clinical and culturally disimilar populations and implementing non-traditional interventions. Andrews et al. show that a single bout of moderate-intensity aerobic exercise lasting 20 min improved motor skill acquisition in individuals with Huntington's disease gene expression. These novel findings are the first step in bridging the gap between animal models and human research, and provide direction for future longitudinal clinical trials of exercise to improve function in people with premanifest and early Huntington's disease. Continuing the theme of investigating the effect of single bouts of exercise, Wen et al. utilized slacklining, a task requiring participants to maintain their balance on a tightened polyester band, somewhat similar to walking a tight-rope. Their young cohort, who were enrolled in college in Taiwan, showed improved inhibitory control with no changes in reaction time following a 50-min bout of slacklining. Understanding the effects of these non-traditional exercise modes are essential for the widespread uptake and individualization of exercise prescription for brain and cognition. Of course, studies like these also highlight the need to better understand the mechanisms by which exercise interacts with the brain. Exercise likely benefits the brain through the physiological response to movement and ensuing cellular and molecular changes (Stillman et al., 2016). With an improved understanding of these mechanisms, it may be possible to move to a model where we focus less on what type of exercise is best and more on which physiological response is required when recommending movement for the brain.

Emphasizing the breadth of research in the field, the final two manuscripts in this Research Topic examine aspects of human performance. Wu et al. examine the effects of training status on changes in neurophysiological markers of brain function following an acute bout of moderate-intensity dragonboat training. The results of this study showed benefits to the functional status of the brain from chronic training and an acute bout of exercise, providing further evidence of the benefits of engagement in exercise, even in a non-traditional modality like dragon boating, for brain health. Finally, Van Cutsem and Pattyn contribute a literature review on the role of sleep in moderating adaptations to altitude training in athletes. In their discussion, they propose a rationale for the role of sleep, specifically the sustained negative impact of altitude exposure on nocturnal breathing rate as an important explanatory variable for variability in adaptations. These two studies, whilst contributing to their discrete areas, further highlight the importance of considering the role of the brain and central processes in maximizing human performance.

## Concluding remarks

This Research Topic illustrates the wide range of research being done to better understand the complex relationship between physical activity and the brain. It demonstrates both the benefits to health and the role of central processes in optimizing physical performance. This diversity in approaches will continue to offer challenges in aligning the field regarding methodological and theoretical approaches. As editors of this Research Topic, we emphasize the importance of multidisciplinary teams combining expertise in human movement and brain health, and the need to better understand mechanisms to explain and predict this relationship.

## Author contributions

JN: Writing – original draft, Writing – review & editing. CH: Writing – original draft, Writing – review & editing. SS: Writing – original draft, Writing – review & editing. KM: Writing – original draft, Writing – review & editing.

## References

[B1] Stillman C. M. Cohen J. Lehman M. E. Erickson K. I. (2016). Mediators of physical activity on neurocognitive function: a review at multiple levels of analysis. Front. Hum. Neurosci. 10:626. 10.3389/fnhum.2016.00626 28018195 PMC5161022

